# Main challenges in developing biotechnology industry in Malaysia: perspectives from the innovative biotechnology firms

**DOI:** 10.1186/1471-2458-12-S2-A25

**Published:** 2012-11-27

**Authors:** Gulifeiya Abuduxike, Syed Mohamed Aljunid, Saperi Sulong

**Affiliations:** 1 United Nation University-International Institute for Global Health, Universiti Kebangsaan Malaysia Medical Centre, Jalan Yaacob Latif, 56000 Kuala Lumpur, Malaysia; 2 Department of Health Information, Universiti Kebangsaan Malaysia Medical Centre, Jalan Yaacob Latif, 56000 Kuala Lumpur, Malaysia

## Background

Innovative biotechnology firms play major roles in the development of biotechnology industry because of their product innovations. Since 2005, the Malaysian government has increased support towards the biotechnology sector to drive this industry forward. The aim of this paper was to identify the challenges in developing biotechnology industry from the prospective of private biotechnology firms in Malaysia.

## Materials and methods

Mixed method approach was applied in the study which consisted of 16 semi structured interviews with key informants from companies and an expert group discussion among industry leaders from private sectors. Qualitative information from these sources was integrated with the information from the secondary sources such as government official reports, published documents and the company websites. Only the companies that applying modern biotechnology to produce health related products were included. Qualitative data was transcribed using Transcriber 5.0 and analyzed with Atlas.ti 6.0 software.

## Results

The study results present that 1) Lack of funding was the most common challenge for all the small medium startup companies. 2) Lack of human capital and essential skill sets was the major barrier for this knowledge driven industry to growth. 3) Poor linkages/interactions between public and private sectors was the main reason for the huge gap in knowledge creation, dissemination across sectors and the root of the conflicts in identifying niche areas.4) Lack of clear regulatory paths/ guidelines for the companies to register their products to access the local market. 5) Lack of knowledge on biotechnology and related aspects among government officers, decision makers was one of the crucial factors to build an effective regulatory system to support the growth of biotechnology companies.

## Conclusions

Biotechnology in Malaysia is facing many critical challenges such as lack of effective financial mechanisms, essential skills set, clear regulatory pathways and so on. Thus, building of an effective national innovation system and prioritizing the basic research science can be key solutions for sustainable development of biotechnology industry.

